# PLLA/GO Scaffolds Filled with Canine Placenta Hydrogel and Mesenchymal Stem Cells for Bone Repair in Goat Mandibles

**DOI:** 10.3390/jfb15100311

**Published:** 2024-10-20

**Authors:** Thamires Santos-Silva, Inácio Silva Viana, Andrea Barros Piazzon S. Queiroz, Fabrício Singaretti de Oliveira, Bianca de Oliveira Horvath-Pereira, Leandro Norberto da Silva-Júnior, Michelle Silva Araujo, Paulo Alescio Canola, Luís Gustavo Gosuen G. Dias, Marcelo Melo Soares, Maria Angelica Miglino

**Affiliations:** 1 Department of Surgery, School of Veterinary Medicine and Animal Science, University of São Paulo, São Paulo 05508-270, SP, Brazil; thamiresssilva@usp.br (T.S.-S.); horvath@usp.br (B.d.O.H.-P.); leandronorberto@unimar.br (L.N.d.S.-J.); msa.vet@hotmail.com (M.S.A.); 2 Department of Veterinary Clinic and Surgery, School of Agricultural and Veterinary Sciences, São Paulo State University, Jaboticabal Campus 14884-900, SP, Brazil; inacio.viana@unesp.br (I.S.V.); paulo.canola@unesp.br (P.A.C.); gustavo.gosuen@unesp.br (L.G.G.G.D.); 3 Department of Animal Morphology and Physiology, School of Agricultural and Veterinary Sciences, São Paulo State University, Jaboticabal Campus 14884-900, SP, Brazil; andrea.queiroz@unesp.br (A.B.P.S.Q.); fabricio.singaretti@unesp.br (F.S.d.O.); 4 Institute of Orofacial Osteogenesis Rehabilitation S/S Ltda., Vila Olímpia 04532-060, SP, Brazil; marcelomelo61@gmail.com; 5 Department of Animal Anatomy, University of Marilia, Mirante, Marília 17525-902, SP, Brazil

**Keywords:** bone defect, computed tomography scans, decellularization, biomaterials, tissue engineering

## Abstract

Bone defects in animals can arise from various causes, including diseases, neoplasms, and most commonly, trauma. Comminuted fractures that exceed the critical size may heal poorly due to deficient or interrupted vascularization, resulting in an insufficient number of progenitor cells necessary for bone regeneration. In this context, 3D printing techniques using poly-L-lactic acid/graphene oxide (PLLA/GO) aim to address this issue by creating customized scaffolds combined with canine placenta hydrogel and mesenchymal stem cells for use in goat mandibles, compared to a control group using titanium plate fixation. Ten canine placentas were decellularized and characterized using histological techniques. A hydrogel derived from the canine placenta extracellular matrix (cpECM) was produced to improve cell attachment to the scaffolds. In vitro cytotoxicity and cell adhesion to the cpECM hydrogel were assessed by scanning electron microscopy (SEM). The resulting biomaterials, cpECM hydrogel and PLLA/GO scaffolds, maintained their functional structure and supported cell adhesion, maintenance, and proliferation in vitro. Thermography showed that PLLA/GO scaffolds with cpECM hydrogel performed effectively, similar to the control group. Computed tomography scans revealed bone calluses, suggesting an ongoing repair process. These findings demonstrate the innovative technological potential of these materials for use in surgical interventions. Future studies on PLLA/GO scaffolds will provide further insights into their effects on goat models.

## 1. Introduction

Bone defects in animals can result from various factors, such as diseases, neoplasms, and most commonly, trauma [1,2,3,4]. These conditions can cause significant damage to the affected region, ranging from simple to complex fractures depending on the force of the impact, and can negatively affect the patient’s quality of life [5].

Under healthy conditions, bone can regenerate without forming scar tissue [6,7]. However, complications arise with impairments, such as comminuted fractures larger than the critical size, delayed healing, poor vascularization or an insufficient number of progenitor cells needed for new bone formation. These complications can lead to non-union of the fracture or even non-healing due to instability in the repair zone [8].

Current treatments for unconsolidated bone fractures (pseudoarthrosis) involve stabilizing the area and often require bone grafting and stimulation of vasculogenesis and osteogenesis [9,10,11,12]. However, these methods often lead to several complications, such as morbidity at the harvest site, deformities, local hematoma, remodeling issues of the implanted bone, and high costs [13,14,15]. Therefore, developing new and effective treatment approaches is essential [7].

Developing new effective treatment approaches, such as bone tissue engineering (BTE), is essential for creating materials that aid in consolidating and repairing damaged bones. The literature comprehensively describes the use of biomaterials that can be combined with cells from various origins to stimulate cell production [14,16,17]. These materials can also be associated with hydrogels that provide gelling properties, such as alginate (a natural polysaccharide extracted from brown seaweed), which is biocompatible, biodegradable, and capable of forming gels under physiological conditions [18,19]. Additionally, new 3D printing techniques have been used to develop customized, personalized, and biomimetic scaffolds [20,21,22,23].

Recent studies have demonstrated that poly-L-lactic acid (PLLA)/graphene oxide (GO) scaffolds, when combined with dental pulp cells and mesenchymal stem cells, exhibit excellent cell adhesion and proliferation [23,24]. The physical and mechanical properties of GO, enhanced by its surface functional groups, are highly desirable for cell growth and regeneration due to its excellent hydrophilicity and adsorption capacity [25,26]. PLLA has been increasingly used in tissue engineering, because its adaptable mechanical properties and biodegradability make it suitable for supporting structures in both in vitro and in vivo applications for new tissue production [27,28,29].

In addition to developing materials that mimic the extracellular matrix (ECM) of bone, selecting an appropriate animal model is crucial to ensure the validity of the study. In this research, goats were chosen because their weight and limb dimensions are comparable to those of humans [30]. Moreover, goats have a similar bone mineral composition, bone remodeling capabilities, and adaptability to temperature changes, make them an excellent model for studying bone defects in the femur, tibia, and radius [30,31,32,33].

This study investigated the effects of implanting PLLA/GO scaffolds filled with ECM from canine placenta and enriched with mesenchymal stem cells into goat mandibles. The goal was to determine whether these scaffolds improve bone recovery and regeneration compared to the control group, which underwent titanium plate fixation.

## 2. Materials and Methods

### 2.1. Ethics Committee

This study was approved by the animal ethics committees of the Faculty of Veterinary Medicine and Animal Science of the University of São Paulo (n° 9130071019) and São Paulo State University, Jaboticabal campus (n° 2689/21).

### 2.2. Study Site and Animals

Nine adult male goats were used and kept at the Central Bioterium of São Paulo State University (Jaboticabal/SP) until the conclusion of the study. The goats were divided into three experimental groups: Group 1 (15 days, *n* = 3), Group 2 (45 days, *n* = 3), and Group 3 (60 days, *n* = 3). Each animal received two implants: a PLLA/GO scaffold with MSCs was implanted on the right antimere of the mandible, and an autologous bone fixed with a titanium plate was implanted on the left antimere. No defects were left untreated. Animal handling and feeding followed standard bioterium protocols.

### 2.3. Decellularization Process and Production of Hydrogel Derived from Canine Placental Extracellular Matrix (cpECM)

All decellularization steps were conducted in sterile conditions under immersion and orbital agitation, according to Matias et al. [34]. Placental samples were collected from neutering campaigns at the Centro Universitário de Jaguariúna (Unijaf), Jaguariúna-SP. A total of 10 placentas (leafy chorion of the zonary placenta) were collected from two pregnant mixed-breed animals. After collection, the placentas were placed on ice and transported to the laboratory, where they were washed with 1% EDTA (LGC Biotecnologia Ltda, Cotia, SP, Brazil), followed by phosphate-buffered saline (PBS; 136.9 mM NaCl, 26.8 mM KCl, 14.7 mM KH_2_PO_4_, pH 7.2). This washing procedure was repeated three times, each lasting for 15 min. Decellularization was performed using a 0.1% sodium dodecyl sulfate (SDS) solution prepared with autoclaved distilled water in a sterile laminar flow hood (No. 13.1313-01; LGC Biotecnologia Ltda, Cotia, SP, Brazil) for 15 days at room temperature. Daily detergent changes were performed within the laminar flow hood. The process concluded with two washes using 1% Triton X-100 (N° 13.1313-01; LGC Biotecnologia Ltda, Cotia, SP, Brazil) under sterile conditions, repeated three times for 15 min each, followed by washes with PBS. This research adhered to the institutional ethics committee guidelines (N° 4959310120).

The hydrogel was produced using a protocol adapted from Matias et al. [34]. For every 10 mg of placenta, 1 mg/mL of porcine pepsin (Sigma, St. Louis, MO, USA) and 0.01 N HCl were added, and the mixture was agitated at 200 rpm at room temperature for 72 h. Following digestion, the canine placenta was neutralized by adding 0.1 N NaOH (1/10 of the pre-gel solution volume) and 10 × PBS (1/9 of the pre-gel solution volume) at 4 °C. Hydrogel polymerization was initiated by adjusting the pH to 7.0 and incubating the solution at 37 °C to allow gelation.

### 2.4. Characterization of cpECM Scaffolds and Hydrogel

#### 2.4.1. Histological Analysis

Fragments of native and decellularized placenta were fixed in 4% paraformaldehyde for 48 h and then dehydrated in a progressive series of ethanol concentrations (70–100%) for 30 min each. Next, the samples were embedded in xylene (I and II) and paraffin (I and II) for 1 h each. Sections were cut at 5 µm using a microtome (LEICA RM 2065) and stained with hematoxylin and eosin (H&E), Masson’s trichrome, Alcian blue, and Picrosirius. The slides were analyzed under a light microscope (FV1000 Olympus IX91, Tokyo, Japan) at the Advanced Image Diagnostic Center (CADI-FMVZ/USP).

#### 2.4.2. Fluorescence Staining Using DAPI (4′,6′-Diamino-2-Phenylindole)

Frozen samples stored at −150 °C were embedded in OCT compound (Tissue Tek O.C.T.) and sectioned to 9 µm thickness using a cryostat (LEICA CM1860, Leica Biosystems, Richmond, IL, USA) at −30 °C. Next, the slides were thawed at room temperature for 15 min and stained with a DAPI solution (1:10,000 in 1X PBS) in the absence of light for an additional 15 min. After staining, the slides were washed with 1X PBS and visualized using a fluorescence microscope (NIKON Eclipse 80i, Nikon Instruments Inc., Melville, NY, USA).

#### 2.4.3. Scanning Electron Microscopy

Small fragments of the samples were fixed in 4% paraformaldehyde diluted in phosphate buffer for 24 h. Subsequently, the samples were washed three times in a sonication bath with distilled water (Unique, USC1450) for 5 min per wash. Next, the samples were dehydrated through a series of increasing ethanol concentrations (70–100%) for 10 min at each concentration. After dehydration, the samples were placed in a critical point dryer for 40 min. The dried samples were mounted on stubs using double-sided carbon tape. For metallization, a thin layer of gold (20–30 nm thick) was applied onto the samples using a sputter coater (# K550-Emitech, Ashford, UK). The samples were observed using a scanning electron microscope (LEO 435 VP^®^, CAE^TM^, New York, NY, USA).

#### 2.4.4. Genomic DNA Quantification

Genomic DNA was isolated from approximately 48–52 mg of native and decellularized canine placentas (processed with 0.1% SDS) using the Illustra^TM^ Tissue and Cells Genomic Prep Mini Spin kit (GE Healthcare, SP, Brazil), following the manufacturer’s instructions. Samples were digested with proteinase K and the kit’s lysis buffer at 56 °C for 3 h. Subsequent processing followed the kit protocol. Purified gDNA was quantified using a spectrophotometer (Nanodrop—Thermo Scientific, Waltham, MA, USA) at 260 nm. DNA concentrations were normalized to the initial tissue mass (48–52 mg). For statistical analysis, the mean ± SD and the *t*-test were calculated.

#### 2.4.5. Hydrogel Toxicity, Adhesion, and Proliferation Assay

To assess the biocompatibility of the cpECM hydrogel, 200 µL of the hydrogel solution was added to culture plates containing complete medium (45 mL of αMEM medium + 5 mL of fetal bovine serum + 1 mL of penicillin-streptomycin + 0.5 mL of amphotericin). The plates were incubated at 37 °C with 5% CO_2_ for 72 h to check for contamination. After this period, 2 × 10^6^ goat mesenchymal stem cells (gMSCs) derived from adipose tissue were pipetted onto the plates for the adhesion and proliferation assay. The cultures were further incubated for seven days.

#### 2.4.6. Total Protein Quantification of cpECM Hydrogel by Bradford Method

Total protein concentration in the cpECM hydrogel was quantified using the Bradford assay kit (B6916; Bio-Rad Laboratories, Hercules, CA, USA). Absorbance of Coomassie Brilliant Blue (CBB) G was measured at a wavelength of 595 nm. Samples were digested in a 1:1 ratio with RIPA (radioimmunoprecipitation assay) buffer solution and incubated for 15 min. Three sample types were evaluated: cpECM hydrogel, 5% alginate, and cpECM hydrogel + 5% alginate. After incubation, the samples were centrifuged at 14,000× *g* for 10 min at 4 °C, and the supernatant was collected and stored at −20 °C until analysis. Protein concentrations were quantified using a standard curve (r^2^ = 0.9975) of 1X BSA (3.2 μg/μL) with concentrations of 0, 0.2, 0.4, 0.8, 1.6, and 3.2 μg/μL. Measurements were performed using a plate reader (UQuant, BioTek, SP, Brazil) with 96-well plates.

#### 2.4.7. Production of (PLLA-GO) Scaffolds

The production, modeling, characterization, and printing of the PLLA-GO scaffolds were described in a previous study [23]. Briefly, GO powder was produced by chemical exfoliation of graphite (Nacional de Grafite Ltda^®^, SP, Brazil) following the modified Hummers method [35]. PLLA (Evonik RESOMER L 210 S) was added to the GO at a concentration of 0.2% of the total polymer weight. The PLLA/GO mixture was prepared at the Mackgrape Laboratory at Mackenzie Presbyterian University (São Paulo, Brazil) and extruded using a standard screw extruder (Thermo Fisher Scientific, Karlsruhe, Germany, Process L/D 40 n° 11). Filaments were cooled on a ventilated conveyor with controlled winding tension to obtain filaments of 1.75 mm thickness. They were then stored in a dehumidifier until 3D printing.

Digital Imaging and Communications in Medicine (DICOM) files were acquired from computed tomography (GE ACTS 16/32, GE Healthcare, Chicago, IL, USA) at a resolution of 0.5 mm using volumetric scanning. The field of view for the specific region was set at a slice thickness of 6 mm, with an image matrix of 512 × 512 pixels of the goat mandibular bone. The DICOM files were converted to standard triangle language (STL) files and used to plan the osteotomy and create a critical defect in the mandibular angle (between the body and ramus of the mandible) using DDS Surgery software (version 2.22.0.2024, JST sp. z. 0.0. μL. Wały Dwernickiego 43/45 42-200, Częstochowa, Poland-CE 0197). Subsequently, a new STL file containing the dimensions of the mandible (4.5 × 3.0 cm) was generated. This file was exported to Blender software (version 2.79b, Blender^®^, Amsterdam, Netherlands) for modeling the osteotomy guide. The files were then transferred to an FDM printer for slicing and modeling and the osteotomy guides were printed in acrylonitrile butadiene styrene (ABS).

#### 2.4.8. Surgical Procedure

The goats were fasted from solid food for 12 to 18 h and from water for 8 to 12 h to prevent aspiration, as fasting helps preserve functional residual lung capacity in ruminants. The goats were positioned in dorsal recumbency, and trichotomy along with aseptic preparation was performed on the right or left jugular groove (the intersection between the omotransverse and brachycephalic muscles), as well as in the submandibular and parotid regions. Venous access to the right or left external jugular vein was established aseptically using an appropriately sized catheter for each animal.

Following catheter fixation and heparin administration, 2% xylazine hydrochloride was administered intravenously at a dose of 0.05 mg/kg. Five minutes later, 0.6 mg/kg of midazolam was administered. Anesthesia was induced with a propofol infusion, using a dose–response effect up to a maximum of 6.0 mg/kg, until orotracheal intubation was achieved with an appropriately sized tube appropriate for each animal.

After intubation and verification of the endotracheal tube position, the volatile anesthetic isoflurane was administered. During the surgery, a continuous infusion of lidocaine (2.0 mg/kg bolus over 15 min) was maintained at an infusion rate of 100 mg/kg/min, ensuring the total dose did not exceed 6 mg/kg/h. Anesthetic monitoring was performed using a multiparameter monitor throughout the surgical procedure.

Following aseptic preparation of the mandibles, the mandibular ramus was accessed through a risk incision, with dissection and retraction of the facial artery. The periosteum was incised at the lower border of the mandible and reflected by blunt dissection, exposing the posterior region of the mandibular body, the mandibular angle, and the inferior portion of the ascending ramus. A previously printed ABS surgical guide was used to remove a bone fragment measuring 3.0 × 2.0 × 0.5 cm (length × height × width) using a reciprocating saw (Osteopower, Osteomed, SP, Brazil). Careful dissection was performed along the intraosseous pathway of the inner mandible.

**Figure 1:**
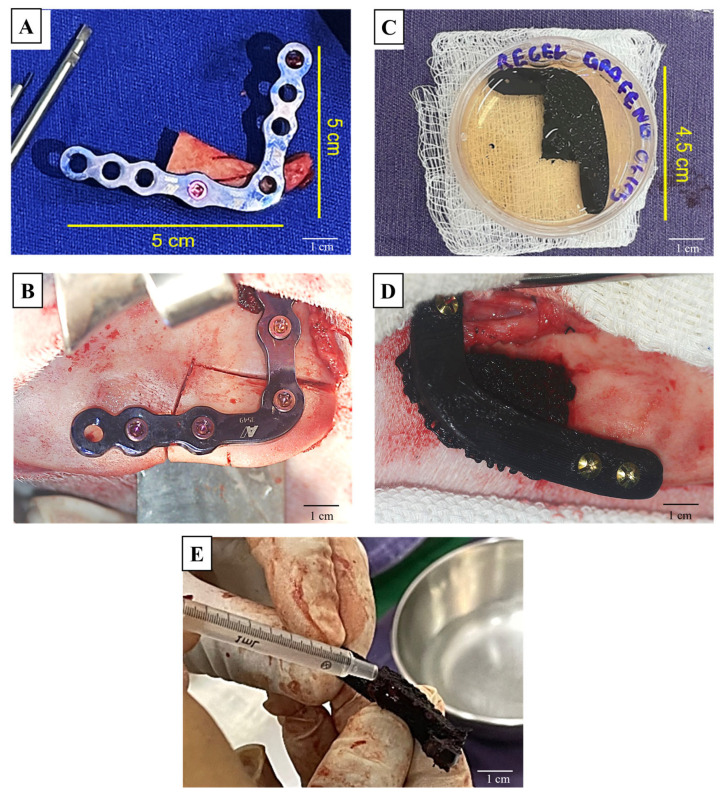
Surgical procedure for implanting PLLA/GO scaffolds and a titanium plate in the goat mandible. Titanium plate fixed to the control bone fragment (**A**); relocation of the bone fragment (left antimere of the mandible) (**B**); PLLA/GO scaffold in cell culture medium (**C**); fixation of the material (right antimere of the mandible) (**D**); PLLA/GO scaffold being filled with hydrogel (cpECM) (**E**). Bars: 1 cm.

The bone fragment removed from the left horizontal ramus of the mandible was kept ex vivo for approximately 5 min, then repositioned and fixed to the recipient site with rigid internal fixation using a 5.0 × 5.0 cm reconstruction plate and 2.4 mm Lock screws. This fragment served as an autogenous bone graft for the critical defect repair. The same osteotomy was performed on the right horizontal ramus of the mandible. The PLLA/GO scaffolds, measuring 4.5 × 3.0 × 1.0 cm, were filled with 2 mL of hydrogel (cpECM), and secured with 2.4 mm screws (Figure 1). Reinforced bars with extensions for the fixation of four screws were added.

The surgical wounds were closed using two types of sutures. The muscle planes were sutured with an interrupted simple suture pattern using absorbable monofilament Polyglactin 3-0 (Shalon Medical^®^, GO, Brazil). For the skin, a non-absorbable synthetic suture with an interrupted simple pattern was performed using 4-0 monofilament nylon (Shalon^®^). After surgery, the goats were kept intubated for 10 min to gradually reduce the amount of inhaled anesthetic. Anesthesia recovery was monitored via pulse oximetry, and extubation was performed when oxygen saturation reached ≥98%.

For postoperative analgesia, methadone (0.24 mg/kg intramuscularly) was administered every 12 h for five days. After the designated analysis periods, the animals were euthanized in accordance with Resolution nº 1000 of the Federal Council of Veterinary Medicine, of 11 May 2012.

#### 2.4.9. Thermographic Analyses

The animals were acclimated in an induction room at 24 °C for 15 min before capturing thermographic images. A FLIR^®^ System AB camera (model T300) was used, with the following settings: emissivity (ε) of 0.98, a distance of 0.6 m, a field of view of 25°, relative humidity of 68%, room temperature of 23.6 °C, and reflectance time of 20°. Images were taken to observe heat distribution patterns on both the right and left sides of the mandible.

#### 2.4.10. Quantification of Heat Distribution Patterns

To analyze heat distribution patterns, five images were taken from both the right and left sides of each animal. These images were quantified using ImageJ win64 software (version 1.48). The average area of the implant regions (in pixels^2^) was estimated by following these steps: (1) the image was opened in the software, (2) the Image/Type/8-bit tab was selected, and (3), the Adjust/Threshold/B&W option was chosen within the same tab to calibrate the black and white scale.

**Figure 2:**
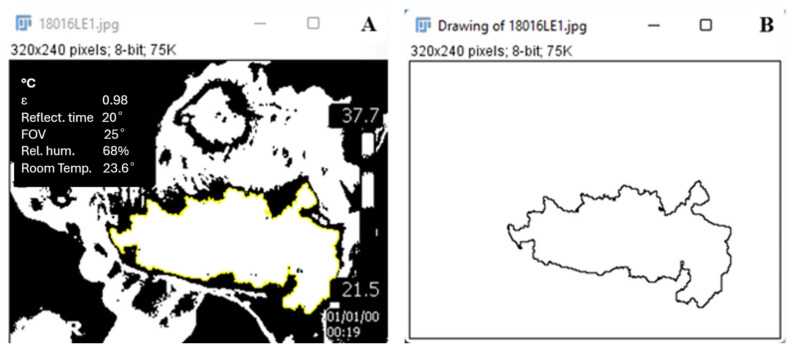
Standardization of images in ImageJ. Image calibrated using the black-and-white scale (**A**); area of interest selected using the wand tool (**B**).

The area of interest was selected using the wand tool, followed by the Analyze/Set Measurement/Area/Standard Deviation/Measure option (Figure 2). The data were tabulated in a Microsoft Excel spreadsheet and analyzed using GraphPad Prism 7.00 software (version 9.5.1). A two-way ANOVA test was conducted with a significance level set at 0.05. Error bars on the graph indicate the minimum and maximum values obtained from the analysis.

#### 2.4.11. Radiographic Analysis of Goat Heads

Computed tomography (CT) and magnetic resonance imaging (MRI) scans were performed using a standard clinical protocol that included 100–140 kV, automatic exposure control, and a slice thickness of 0.625 mm. Bone and soft tissue algorithms were applied using a multidetector tomography machine (SIGNA™ Creator-GE HealthCare^®^, SP, Brazil). The goat heads were positioned in a dorsal/supine decubitus position on the scanning table to obtain transversal and sagittal views. Images were reviewed using the Clinical Image Communication and Archiving System (wetransfer.com), URL (accessed on 2 April 2023).

**Figure 3:**
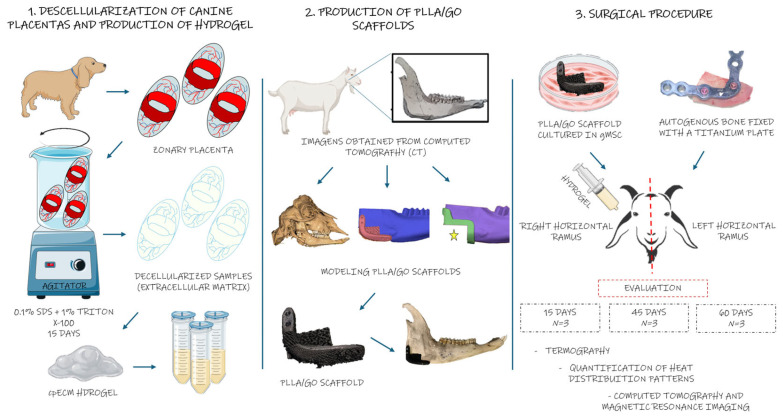
Graphical representation of the experimental process for bone repair in goat mandibles. The image illustrates the steps; from preparation of biological materials to scaffold integration and implantation. The canine placenta is processed into a hydrogel, which is introduced into 3D-printed scaffolds made of PLLA and graphene oxide (GO) and cultured with goat mesenchymal stem cells. These scaffolds are applied to a 3D-modeled mandibular defect in goats, followed by surgical implantation for bone repair, with evaluations conducted at 15, 45, and 60 days.

Figure 3 summarizes the steps performed and illustrates how the components were assembled.

## 3. Results

### 3.1. Evaluation of cpECM Scaffolds and Hydrogel Composition

**Figure 4:**
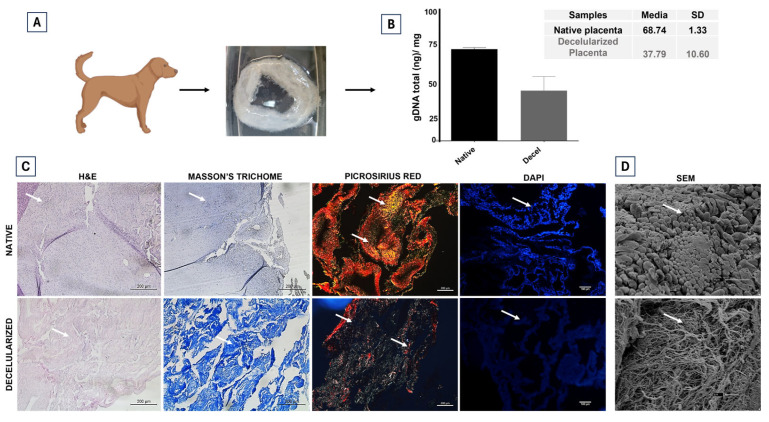
Characterization of canine placenta extracellular matrix (cpECM) scaffolds. (**A**) The decellularized placenta after 15 days. (**B**) DNA content of the native and decellularized placentas. N = 6, *p* < 0.05. (**C**) Representative images showing H&E staining (left panel), followed by Masson’s trichrome, picrosirius red and DAPI fluorescence staining of both native and decellularized canine placenta. The cell nuclei were removed, but the matrix structure was maintained (arrows). Scale bars: 200 μm (H&E; Masson’s Trichome and Picrosirius); Scale bars: 100 μm (DAPI). (**D**) Scanning electron microscopy (SEM) images of native and decellularized canine placenta. Scale bars: 30 μm (native) and 3 μm (decellularized).

After 15 days of decellularization, the canine placenta exhibited a gel-like texture and a translucent appearance (Figure 4A). To assess the reduction in DNA in the material, the average genomic DNA content of native and decellularized canine placentas was quantified. The DNA content decreased from 68.74 to 37.79 ng/mg, which is within the recommended parameter of less than 50 ng/mg of tissue (Figure 4B). Moreover, no obvious nuclear components were observed in the decellularized ECM, as indicated by H&E staining and DAPI fluorescence. Masson’s trichrome staining demonstrated the preservation of the ECM, with the structure of the collagen fibers remaining intact. These fibers were further highlighted by Picrosirius red staining, which revealed mature fibers in red, yellow, and orange, while immature fibers retained after decellularization appeared in greenish shades (Figure 4C).

**Figure 5:**
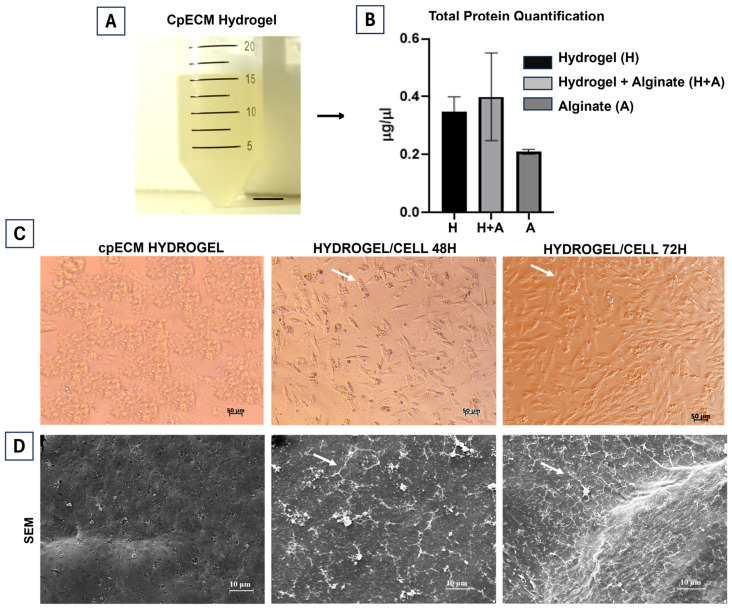
Characterization of the canine placenta extracellular matrix (cpECM) hydrogel. (**A**) cpECM hydrogel. (**B**) Quantification of total protein in cpECM hydrogel, 5% alginate, and their combinations. (**C**) Representative images of hydrogel and hydrogel + MSCs after 48 and 72 h. Scale bars: 50 μm. (**D**) Scanning electron microscopy (SEM) images of hydrogel and hydrogel + MSC after 48 and 72 h. The arrows indicate an increase in the number of cells after 48 and 72 h. Scale bars: 10 μm.

After analyzing the decellularized tissue, the cpECM was fragmented and digested with porcine pepsin to produce a hydrogel. The resulting ECM solution exhibited a gel-like consistency, with no fragments or residual tissue (Figure 5A). The total protein content of the cpECM hydrogel, 5% alginate, and a 1:1 mixture of cpECM and 5% alginate was quantified. The cpECM hydrogel contained 0.35 μg/μL of total protein, the alginate contained 0.21 μg/μL, and the mixture of cpECM and 5% alginate contained 0.40 μg/μL (Figure 5B).

To evaluate the cytotoxicity of the cpECM hydrogel, goat mesenchymal stem cells (gMSCs) were cultured in a two-dimensional setup for 72 h to assess their adhesion capacity. After 48 h, the cpECM hydrogel exhibited a uniformly porous surface that supported cell proliferation and adherence. By 72 h, the cells had proliferated throughout the plate. This proliferation was attributed to the various ECM components, such as growth factors, which bind to the hydrogel proteins, promoting and regulating cell proliferation and migration (Figure 5C). Scanning electron microscopy demonstrated that the hydrogel was biocompatible and supported cell activity, resulting in an increase in cell numbers (Figure 5D).

### 3.2. Post-Surgery Evaluation of Animals

All animals recovered well from the surgery, with satisfactory wound healing, and no clinical signs of pain. Sutures were removed after 15 days, and complete healing was observed by day 20. Thermographic analysis showed that skin temperature regulation, a key function for maintaining body temperature, was effective. In goats, thermoregulation maintains body temperature between 38.5 and 39.7 °C and the animals approached this range in all measurements.

**Figure 6:**
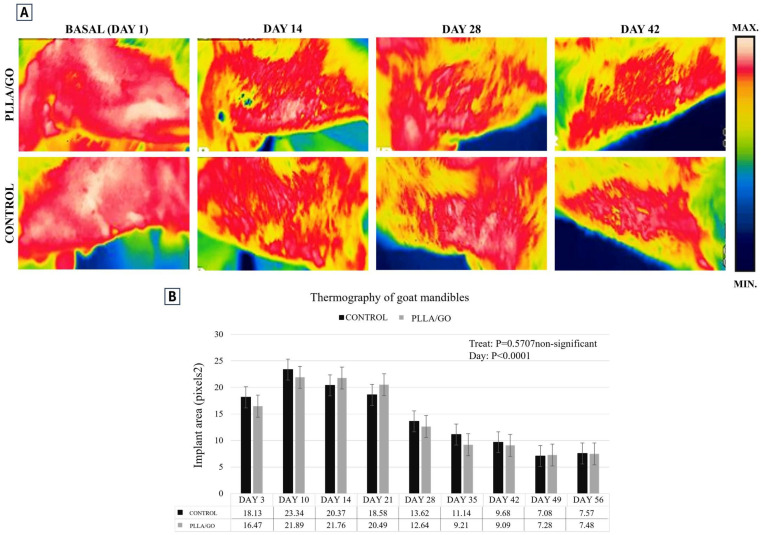
Thermographic images of goat heads. (**A**) The image shows the temperature variation at the mandibular implant region (control and PLLA/GO) on the 1st, 14th, 28th, and 42nd day post-surgery. The red areas represent the highest temperatures, typically indicating increased metabolic activity or inflammation, while the yellow, green, and blue areas correspond to progressively cooler regions. (**B**) The graph represents the mandible implant area over time, showing how the size of the area decreased over the days. It demonstrates no significant difference between the PLLA/GO treatment and the control group up to day 56.

Infrared light detection revealed changes in body temperature due to variations in blood flow, indicating physiological alterations rather than anatomical abnormalities. Thermographic images showed a reduction in temperature at the mandibular implant region (both control and PLLA/GO) on the 28th day post-surgery (Figure 6A). From this day onwards, the temperature decrease was similar on both sides, with minimal difference despite the smaller covered area of the PLLA/GO implants. Although ANOVA showed no significant difference between treatments (control and PLLA/GO), a significant temperature reduction was observed on both sides by day 56 (*p* < 0.0001) (Figure 6B).

**Figure 7:**
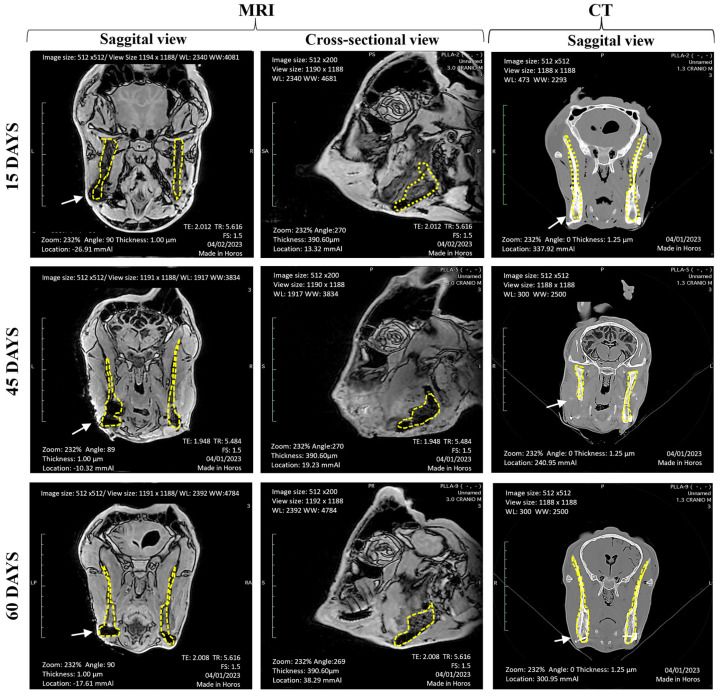
Magnetic resonance imaging (MRI) and computerized tomography (CT) images of goat heads. The yellow dotted lines outline the analysis area, and white arrows indicate the critical defect region with PLLA/GO, with the control on the opposite side. Group 1 (15 days) showed no evidence of change or bone growth. Group 2 (45 days) exhibited a reduction in the critical defect area but no signs of bone growth. Group 3 (60 days) demonstrated bone callus development, as seen in the CT images.

MRI and CT provide valuable information about the size and location of critical defects, as well as their interactions with nearby structures. CT scans revealed details about the mineralization of cortical and trabecular bone-like matrices, whereas MRI offered better contrast between soft tissues and adjacent muscle planes [36]. Figure 7 shows the critical defect areas in all animals, illustrating how PLLA/GO scaffolds replicated trabecular bone architecture. This replication was possible due to the interconnected porous structure of the scaffolds, which facilitates cell migration upon direct contact with bone tissue.

Animals from Group 1 (15 days) showed no obvious structural changes. In contrast, animals from Group 2 (45 days) showed a more favorable response to the material compared to Group 1. Although the defect area decreased in the sagittal view, the osteogenesis rate remained low because of the slow degradation of the PLLA/GO scaffolds which limited space for bone tissue growth. Finally, animals from Group 3 (60 days) showed bone callus formation in the transversal view on CT scans, indicating progress in the repair stage.

Although this study did not quantify the rate of scaffold degradation or the production of new bone tissue, numerous studies have shown that biomaterials can help defective bones adapt to external loads, significantly impacting bone growth capacity [37].

## 4. Discussion

This study examined the effects of implanting PLLA/OG filled with cpECM enriched with mesenchymal stem cells in goat mandibles to determine whether these scaffolds improve bone regeneration and recovery compared to a control group consisting solely of titanium plate fixation, with the aim of evaluating their potential for clinical and surgical applications. To produce the cpECM hydrogel, the native and decellularized materials were characterized. After 15 days of using 0.1% SDS, it was confirmed that this detergent preserved tissue structure while removing cellular material, consistent with previous findings [38].

Other decellularized materials and associated protocols, such as human dermis [39], blood vessels [40], and swine heart valves [41], have been approved for clinical use, similar to canine placenta. These techniques aim to reduce immunogenicity and stimulate the growth of mesenchymal stem cells [42]. H&E, Masson’s trichrome, DAPI, picrosirius red, and Alcian blue staining methods demonstrated that the ECM structure was preserved. The gDNA measurements indicated that the decellularized tissues contained less than 50 ng/mg of DNA, consistent with the literature guidelines [38].

Subsequently, the cpECM hydrogel was developed, providing an optimal environment for anchoring and cultivating gMSC. This finding aligns with the research by Francis et al. [43], who produced an ECM hydrogel from human placenta for heart cell culture with potential therapeutic applications. Although the cell types differ, both studies highlight the clinical relevance of using placenta-derived materials, as the placenta is a temporary organ that is widely available and discarded after birth [44,45].

Another important aspect of hydrogel synthesis is maintaining sterility. The material showed no effect on cell growth after 72 h of culture. This is because the ECM structure and components contain proteins (types I, III, and IV collagen) and adhesion molecules (e.g., laminin) that support cell proliferation [45]. Furthermore, total protein quantification of the hydrogel showed no significant losses, whether used alone, in combination with 5% alginate, or with 5% alginate alone.

The gMSCs derived from adipose tissue used in this study were selected for their potential to stimulate bone formation, given their self-renewal capabilities [46] and tissue-specific differentiation [47,48]. Moreover, these cells have shown promise in both experimental and clinical trials for veterinary and human diseases [49]. The combination of MSCs and PLLA/GO scaffolds enhances bone tissue regeneration, as these scaffolds are mechanically resistant [24] and support cell adhesion and proliferation [23,24,50]. The goal of combining these materials for in vivo investigations was to determine if scaffolds filled with cpECM and cells would promote bone repair. Although immunologic assays will be analyzed in a future study, the polymers (PLLA/GO) have properties such as resistance to cellular degradation, a hydrophilic surface important for polymers used in bone tissue repair [51], and biocompatibility, which has been previously investigated [23].

Moreover, the combination of PLLA and GO provided better control over the mechanical properties of the scaffolds by enabling adjustments in concentrations, cross-linking levels, and copolymerization. This ensures uniform and reproducible properties with a lower risk of infection and immunogenicity [52]. This is supported by a graph showing average heat distribution in goat mandibles. After 28 days post-surgery, the temperature on both sides of the implant decreased without inducing an inflammatory response. Although the PLLA/GO group exhibited a greater reduction in temperature than the control group, the difference was not statistically significant. Qin et al. [53] reported similar findings when testing GO/attapulgite/gelatin scaffolds for healing critical defects in mouse skulls using 3D printing. Their study demonstrated that these scaffolds did not induce an inflammatory response and promoted osteogenic differentiation of MSCs obtained from human adipose tissue without the need for an osteogenic induction medium. This is possible because polymers such as PLLA and GO are projected to be biocompatible, meaning they are less likely to provoke an immune response [54,55], and have adjustable mechanical properties. The addition of GO to other materials improves tensile resistance, making them more similar to natural bone tissues [56]. Moreover, GO can modulate biological signals through interactions with cells and tissues, facilitating cell adhesion, proliferation, and differentiation, which aids in bone regeneration [57]. Additionally, design flexibility is possible, as evidenced by previous studies that developed customized PLLA/GO scaffolds for bone repair [23]. This flexibility is particularly advantageous for complex bone repair surgeries or when addressing significant bone tissue loss or damage.

In contrast, there is limited research on MRI and CT for evaluating bone and biomaterials in animals. For instance, Jones et al. [58] investigated bone integration in porous biomaterials in vitro using RM and showed that bone growth primarily occurs at the periphery of the structure, with a gradual decrease in mineralization in volume. Although pore size was not evaluated in the present study, similar results were observed in previous research on PLLA/GO characterization [23]. Vasconcellos et al. [59] used micro-CT to examine bone integration in porous titanium implants. Their study included both morphological and qualitative analyses of bone formation around biomaterials, revealing that porosity and pore size positively influence bone growth. Nevertheless, PLLA/GO biomaterials have shown promise in various biological applications, including tissue regeneration and engineering. However, challenges and limitations regarding their compatibility and safety have been discussed in the literature [60,61].

One key challenge with PLLA is controlling its degradation rate. Gradual degradation is desirable for promoting tissue regeneration, but if it occurs quickly, it can lead to the premature release of degradation products, potentially causing inflammation and exacerbating the immunological response. Conversely, if degradation is slow, the implant may persist in the body longer than intended, increasing the risk of long-term complications such as fibrous encapsulation or foreign body formation [62,63].

Regarding GO biocompatibility and safety, further investigation is needed. Although GO exhibits unique mechanical and electrical properties that can facilitate integration between the implant and surrounding tissue, concerns remain about its cytotoxicity and pro-inflammatory potential. Research suggests that GO particles may induce an inflammatory response, potentially leading to increased scar tissue formation and challenges with implant integration. Moreover, the long-term effects of the migration of GO particles to other tissues and organs are undesirable [64,65,66,67].

In earlier investigations, the biological behavior of PLLA/GO composites was explored, revealing that gMSCs exhibited the best adherence to various composite trabeculae, with an elongated morphology similar to fibroblasts. The distribution of filaments during cell seeding had minimal impact on culture efficiency [23,24]. Therefore, it is essential to conduct further assays and develop design and fabrication procedures that enhance clinical efficacy while minimizing the risks associated with these biomaterials. To better understand the influence of these scaffolds on the goat organism, it is necessary to evaluate the immune response, integration with native bone, and hydrogel capacity in combination with MSCs to promote the development of new bone tissue.

## 5. Conclusions

After 15 days, the decellularization process using 0.1% SDS successfully produced decellularized placentas, as confirmed by histological analysis, DNA quantification, and scanning electron microscopy. The resulting biomaterials cpECM hydrogel and PLLA/GO scaffolds maintained their integrity structural and supported cell adhesion, maintenance, and proliferation in vitro. Thermographic analysis indicated that the PLLA/GO scaffolds and cpECM hydrogel performed similarly to the control, demonstrating their potential as novel surgical technologies. Future studies on PLLA/GO scaffolds will provide further insights into their effects on goat models.

## Data Availability

The original contributions presented in the study are included in the article, further inquiries can be directed to the corresponding author/s.
